# Bile acids accumulate norovirus-like particles and enhance binding to and entry into human enteric epithelial cells

**DOI:** 10.1128/jvi.00342-26

**Published:** 2026-05-06

**Authors:** Elin Hahlin, Katarina Danskog, Stefan Nord, Miriam Becker, Stefanie M. A. Willekens, Carin Wibom, Hugh Tanner, Lars Frängsmyr, Daniel Öhlund, Linda Sandblad, Annasara Lenman, Niklas Arnberg

**Affiliations:** 1 Department of Clinical Microbiology, Umeå University, Umeå, Sweden; 2 Swedish Defense Research agency, CBRN, Defense and security, Umeå, Sweden; 3 Institute for Experimental Virology, TWINCORE, Centre for Experimental and Clinical Infection Research, a joint venture between the Medical School Hannover and the Helmholtz Centre for Infection Research, Hannover, Germany; 4 Department of Biochemistry & Research Center for Emerging Infections and Zoonoses (RIZ), University of Veterinary Medicine Hannover 26556 , Hannover, Germany; 5 Wallenberg Centre for Molecular Medicine (WCMM), Umeå University, Umeå, Sweden; 6 Umeå Centre for Microbial Research (UCMR) 504517 , Umeå, Sweden; 7 The Laboratory for Molecular Infection Medicine Sweden (MIMS), Umeå University, Umeå, Sweden; 8 Umeå center for Functional Brain Imaging (UFBI), Umeå University 8075, Umeå, Sweden; 9 Department of Chemistry, Umeå University, Umeå, Sweden; 10 Department of Diagnostics and Intervention, Umeå University, Umeå, Sweden; 11 Science for Life Laboratory (SciLifeLab) 463758, Umeå, Sweden; University of Kentucky College of Medicine, Lexington, Kentucky, USA

**Keywords:** cell entry, gastroenteritis, bile acid, bile, norovirus

## Abstract

Human norovirus (HuNoV) is a leading cause of acute viral gastroenteritis, but despite high impact on public health and healthcare, the mechanisms of viral attachment to and entry into target cells are not yet fully understood. Recent reports indicate that saliva and bile contribute to the transmission of HuNoV. For example, human bile acids increase cell surface ceramide levels in human enteroids, which improves norovirus entry into cells, resulting in enhanced replication. Bile acids can also interact directly with the norovirus capsid, but it is not known whether bile or other gastrointestinal body fluids directly affect HuNoV attachment to host cells. In this study, we investigated the effects of patient-derived gastric juice, pancreatic juice, and bile on HuNoV GII.4 virus-like particle (VLP) attachment to and entry into a human duodenal cell line, HuTu-80. We show that while gastric juice and pancreatic juice do not affect viral attachment or entry, bile—in particular, hydrophobic bile acids—significantly enhance cellular attachment and subsequent entry of GII.4 VLPs into cells. In addition, we show that hydrophobic bile acids induce the accumulation of viral particles in the vicinity of cells. These results suggest the presence of a new *en masse* infection mechanism, where bile acids aggregate virions and allow direct and more efficient attachment to and entry into target cells.

**IMPORTANCE:**

Viruses transmitted by the fecal-oral route encounter secreted host factors in gastrointestinal fluids. Some host factors can be exploited by the virus to facilitate infection. Human bile acids indirectly promote norovirus entry into and infection of human enteroids, but the direct effect of bile acids on attachment and uptake, along with the impact of other gastrointestinal fluids, remains unknown. Here, we investigated the direct effects of human body fluids on cellular attachment of norovirus VLPs. We show that human bile and hydrophobic bile acids induce an accumulation of norovirus VLPs, which is associated with significantly enhanced attachment and entry into human duodenal cell lines. These results highlight the differential effects of gastrointestinal body fluids on viral attachment and entry while providing useful information on the complex HuNoV-host interactions that facilitate infection.

## INTRODUCTION

Human norovirus (HuNoV) is one of the most common causes of acute viral gastroenteritis worldwide. It is estimated to cause over 200,000 deaths annually and constitute a global economic burden of more than $60 billion (1). Noroviruses are non-enveloped positive-sense RNA viruses belonging to the *Caliciviridae* family. They are classified into 10 different genogroups (GI–GX), which are further divided into 48 genotypes. Viruses belonging to genogroups I, II, IV, VIII, and IX infect humans (2, 3), and out of these, genotype GII.4 is currently the most common cause of acute infection (4–6). The genome is contained within a shell of major capsid protein VP1 and minor capsid protein VP2, where VP2 is located inside the capsid to increase stability. VP1 consists of a shell (S) and a protruding (P) domain (7, 8), where the P domain is further subdivided into a P1 and a receptor-interacting P2 domain. The P2 domain is the least conserved and contains sites that contribute to cell binding, antigenicity, and immune-driven evolution (9). Expression of VP1 in the baculoviral-expression system results in self-assembly of virus-like particles (VLPs) that are comparable to the native virus in size and appearance (10).

To date, there are no available vaccines or antivirals, partly due to HuNoV research being hampered by the, until recently, lack of a working cell model to cultivate the virus (3, 11–15). Research on HuNoV has thus resorted to using alternatives such as virus-containing stool samples and VLPs that can bind and enter cells but are unable to replicate (16, 17). Murine norovirus (MNoV) is a good model for HuNoV as it readily replicates *in vitro,* but it does not cause severe gastrointestinal disease in adult immunocompetent mice (18). However, specific strains of MNoV, such as MNoV-3, can cause gastrointestinal symptoms in interferon-deficient STAT1^-/-^ mice (19) and MNoV-1 in neonatal BALB/c mice (20, 21). Susceptibility to HuNoV infection is associated with expression of histo-blood group antigens (HBGAs) on epithelial cells (16, 17). Most GII.4 and GII.3 genotypes are HBGA-binders, whereas GII.1 and GII.2 mainly contain non-binders (22). HBGA expression is mediated by the enzyme fucosyltransferase-2 (FUT2), which adds a fucose residue to glycan chains on the cell surface, structures that the P2 domain of VP1 recognizes and binds (16, 23–25). Fucose thus functions as an attachment factor, and so-called secretor-negative individuals, lacking a functional fucosyltransferase-encoding *FUT2* gene, are highly resistant to infections with HBGA-dependent norovirus strains (26, 27). However, some studies have found that secretor-negative individuals can be infected by HBGA-dependent HuNoV genotypes (28–30). More recently, it was demonstrated that the S domain of VP1 binds to galectin 3 and apoptosis-linked gene 2-interacting protein X, which can facilitate entry of GII.4 VLPs (31). It is also known that human tissue fluids contain components that affect virus attachment to target cells. Coagulation factors and lactoferrin, for instance, can facilitate non-canonical cellular attachment and entry of human adenovirus (32–34), while host trypsin-mediated cleavage of influenza A hemagglutinin is essential for membrane fusion (35, 36). Similarly, MNoV can replicate in salivary glands and be transmitted through saliva (37). GII.4 HuNoV can also replicate in a salivary cell line, but whether saliva contributes to increased transmission is unknown. In the gastrointestinal tract, bile is a body fluid mostly consisting of highly hydrophobic bile acids, synthesized from cholesterol in the liver (38–40). Bile acids have previously been shown to regulate replication of the porcine enteric calicivirus strain Cowden (41) and are essential for successful replication of GII.3 genotypes in human intestinal enteroids (HIEs) (5, 42). Previous studies have shown that the bile acid glycochenodeoxycholic acid (GCDCA) increases the production of cell surface ceramide, leading to enhanced uptake of HuNoV GII.3 in HIEs (42). Furthermore, it was recently found that incubating bile acids with HuNoV GII.4 increases ceramide at the cell surface, which is associated with increased membrane permeability (31). These findings suggest that bile acids enhance HuNoV replication via stimulatory effects on host cells. Still, structural studies have identified bile acid binding pockets in several HuNoV genotypes, including GII.1, GII.4, GII.10, and GII.19, indicating that bile acids may play multiple roles in the HuNoV replication cycle (43, 44). For MNoV, bile acids have been shown to interact directly with the P domain (45). GCDCA specifically enhances binding of MNoV to its proteinaceous receptor CD300lf (46). This interaction involves key residues within the P domain and induces a conformational rearrangement of the entire domain (46, 47).

While it is clear that bile contributes to HuNoV infection, the role of other gastrointestinal fluids, such as gastric and pancreatic juice, has not been investigated in relation to entry. In this study, we collected patient-derived gastrointestinal fluids and investigated their effects on HuNoV GII.4 VLP interactions with the duodenal cell line, HuTu-80. We show that gastric and pancreatic juice does not affect the initial steps of the viral replication cycle. In contrast, bile acids, and specifically hydrophobic bile acids, contribute to the attachment and uptake of GII.4 VLPs into HuTu-80 cells. Additionally, we show that the VLPs form clusters when incubated with bile, or the highly hydrophobic bile acid taurolithocholic acid (TLCA). Several enteric viruses, including HuNoV, can be released within extracellular vesicles containing multiple viral particles, thereby increasing the chance of effective infection both *in vitro* and *in vivo* (48, 49). Recently, it has also been shown that HuNoV GII.4 VLPs can cluster on the cell surface in an HBGA-dependent manner, leading to increased binding and entry in HIEs from the small intestines (50). The bile acid-dependent accumulation of viral particles demonstrated in this study suggests the presence of another potential mechanism used by HuNoV for attachment to, entry into, and productive infection of duodenal cells.

## MATERIALS AND METHODS

### Cells, antibodies, and VLPs

Human duodenal adenocarcinoma cell line HuTu-80 (ATCC #HTB-40) was cultured in Dulbecco’s Modified Eagle’s Medium (DMEM; Sigma-Aldrich), 20 mM HEPES (Sigma-Aldrich), 10% Fetal Bovine Serum (FBS; Cytiva), 100 units/mL of penicillin + 100 µg/mL of streptomycin (PEST, Gibco) in a humidified incubator at 37°C with 5% CO_2_. For VLP detection, the following antibodies were used: MAB227P Monoclonal antibody against GII.4 Norovirus (Mouse IgG2a κ, Clone 2002-G5, Maine Biotechnology Services), monoclonal antibody against GII.1 norovirus mouse monoclonal HNoV GII 2002.G2 (51), rabbit anti-Alexa Fluor 488-conjugated polyclonal IgG (Thermo Fisher Scientific), Alexa Fluor 647-conjugated rabbit anti-mouse polyclonal IgG (Thermo Fisher Scientific), Alexa Fluor 568-conjugated goat anti-rabbit IgG (Thermo Fisher Scientific), Hoechst33342 (Thermo Fisher Scientific), and Alexa Fluor 647-conjugated Wheat Germ Agglutinin (Thermo Fisher Scientific). HuNoV GII.1 (HV strain) (52) VLPs and monoclonal antibody GII.4-2002G2 for GII.1 (clone 66.691.7.2) (51), used for the detection of GII.1 VLPs, were a kind gift from Dr. Lisa Lindesmith.

### GII.4 VLP production

VLPs based on the human norovirus GII.4 strain NLV/DIJON171/96 (GeneBank accession no. AAL79839.1) were produced with the Bac-to-Bac TOPO Expression system (Invitrogen, Catalog nos. A11101, A11100) according to instructions from the manufacturer. Supernatant was collected from infected SF-9 cells, and cellular debris was removed by centrifugation at 3,000 × *g* for 30 min at 4°C. The VLPs were concentrated by high-speed centrifugation at 100,000 × *g* for 2 h at 4°C. The obtained pellet was dissolved in 0.2 M Tris-HCl, pH 7.4, loaded onto a discontinuous sucrose gradient 20%–60% and centrifuged at 175,000 × *g* for 16 h at 4°C. From the sucrose gradient, the band corresponding to intact VLPs was extracted and stored at −80°C until further use. For use in experiments, the buffer was exchanged to 25 mM HEPES, pH 8.2, by use of an Amicon Ultra Centrifugal filter, 100 kDa MWCO, and VLPs were stored at 4°C.

### Human body fluid samples and bile acids

Gastric juice, pancreatic juice, and bile were collected from adults undergoing hepatobiliary surgery at Umeå University Hospital. The study was conducted according to the Declaration of Helsinki and was approved by the Swedish Ethical Review Authority (2019-00399 and 2019-03805). All subjects taking part in the study provided written informed consent. Samples were aliquoted and stored at −80°C until use. Bile acids were purchased from Sigma-Aldrich. GUDCA, GCA, GDCA, GCDCA, TCDCA, TCA, and TDCA were dissolved in ultrapure water, whereas CA, CDCA, DCA, UDCA, LCA, and TLCA were dissolved in dimethyl sulfoxide (DMSO), and cholesterol (Sigma-Aldrich) in ethanol, all to a concentration of 50 mM.

### Binding assay

HuNoV VLP binding was evaluated using flow cytometry. HuTu-80 cells were detached using PBS supplemented with 0.05% EDTA, counted, and reactivated in growth medium for 1 h at 37°C on a tipping table. Cells were plated in 96-well plates (2 × 10⁵ cells/well) and washed with PBS. Meanwhile, HuNoV GII.4 or GII.1 VLPs were preincubated with patient-derived body fluids (bile, pancreatic juice, or gastric juice) diluted in PBS to a concentration of 12.5%, or with pure bile acids/cholesterol (diluted in PBS to 4 mM), for 30 min at 37°C on a rotary shaker. Before addition to cells, the mixtures were further diluted in PBS to a final concentration of 0.78% or 250 µM, respectively, and a VLP concentration of 6 µg/mL. The VLP mixtures were added to cells and incubated for 1 h at 4°C on a rotary shaker, followed by a wash with PBS. For staining, anti-norovirus antibodies MAB227P for GII.4, and mouse 2002.G2 for GII.1, diluted 1:750 and 1:300, respectively, in FACS buffer (PBS with 2% FBS), were added and incubated with the cells for 30 min at 4°C on a rotary shaker. Cells were washed once with FACS buffer, and secondary antibody, rabbit anti-mouse Alexa Fluor 647 (Thermo Fisher Scientific), diluted 1:1,000 in FACS buffer, was added and incubated with the cells for 30 min at 4°C on a rotary shaker. Cells were washed once and analyzed by flow cytometry with a BD Accuri C6 instrument (BD Biosciences).  Gating was done for single cells based on side scatter, and in each sample, 10,000 events were recorded and analyzed for geometric mean fluorescence intensity.

#### Bile acid pre-treatment of cells before VLP binding

Cells were incubated with bile acids for 30 min at 37°C. Supernatant, containing excess bile acids, was removed, and cells were washed once before VLP mixtures were added for 1 h at 4°C on a rotary shaker, followed by staining as described above.

### Cell cytotoxicity assay

HuTu-80 cells were detached using PBS supplemented with 0.05% EDTA, counted, and reactivated in growth medium for 1 h at 37°C on a tipping table. Cells were seeded in white opaque 96-well flat-bottom plates (Grenier) at a density of 2 × 10⁵ cells/well and washed with PBS. Bile acids were diluted in PBS in twofold dilutions from 1 mM to 62.5 µM and preincubated for 30 min before addition to wells in duplicates. The plates were incubated for 1 h at 4°C on a rotary shaker. Cell viability was measured by ATP production using the CelltiterGlo Luminescent Cell Viability Assay kit (Promega), according to the manufacturer’s instructions, and analyzed with a CLARIOstar plate reader (BMG Labtech).

### Cell permeability assay

Cell permeability was evaluated by flow cytometry. Samples were prepared as described in the binding assay, and the positive control was incubated with 0.05% Tween-20. Cells were stained with propidium iodide (PI) diluted 1:5,000 in FACS buffer for 30 min at 4°C on a rotary shaker, washed twice, and analyzed by flow cytometry using a ZE5 instrument (BioRad). Gating was done for single cells based on side scatter, and in each sample, 10,000 events were recorded and analyzed for geometric mean fluorescence intensity of PI.

### Surface plasmon resonance

All measurements between HuNoV GII.4 VLPs and bile acids were performed at 25°C using a Biacore T-200 instrument. VLPs were immobilized onto a CM5 chip, using the Amine Coupling Kit (GE Healthcare), to a concentration of 5–8 ng·mm^2^ (∼5,000 to 8,000 response units; RUs). All binding assays were performed at 25°C using running buffer (10 mmol/L phosphate buffer [pH 7.4], 140 mmol/L NaCl, 0.27 mmol/L KCl, and 0.05% Tween-20). The analytes (bile acids) were serially diluted in running buffer (250, 200, 126, 100, 50, and 25 mM), and then injected in series over the reference and experimental biosensor surfaces for 120 s at a flow rate of 30 µL/min. Blank samples containing only running buffer were injected under the same conditions to allow for double referencing. After each cycle, the biosensor surface was regenerated with a 60-s pulse of 10 mM glycine (pH 1.5) at a flow rate of 30 µL/min.

### Norovirus VLP labeling

HuNoV GII.4 VLPs were labeled with an Alexa Fluor 488 NHS Ester (Thermo Fisher Scientific), dissolved in DMSO at a concentration of 10 µg/mL. Dye was added to purified VLPs (in 25 mM HEPES buffer pH 8.2) at a 10× molar excess while vortexing, and the reaction was left for 1 h at room temperature under constant rotation. Afterward, excess dye was removed by buffer exchange to PBS using filter centrifugation (Amicon, Millipore).

### Entry assay

HuTu-80 cells were seeded in eight-well glass-bottom chamber slides (Nunc, Lab Tek II) at a density of 150,000 cells/well and placed in a humidified incubator at 37°C with 5% CO_2_. The following day, Alexa Fluor 488-labeled GII.4 VLPs (6 µg/well) were pre-incubated with TLCA, GCDCA, GCA, CA, or human bile (sample 3) at a concentration of 4 mM (bile acids) or 12.5% (bile), at 37°C for 30 min and subsequently diluted to 250 µM (0.78% for bile samples) in PBS. VLPs were allowed to bind to the cell surface for 1 h at 4°C. After washing, particles were allowed to internalize for 2 h at 37°C. Binding control samples were kept on ice. All staining was performed on ice. Samples were incubated with blocking buffer (PBS supplemented with 2% BSA) for 15 min and stained for non-internalized virus particles with an anti-Alexa Fluor 488 antibody diluted 1:500 in blocking buffer for 1.5 h, and a secondary Alexa Fluor 568 1:1,000 in blocking buffer for 1 h. Cell nuclei were stained using Hoechst 33342 1:5,000, and Alexa Fluor 647-conjugated Wheat Germ Agglutinin 1:500 diluted in blocking buffer was used for plasma membrane visualization. Samples were fixed with 4% PFA and imaged with a Leica SP8 Confocal microscope at a magnification of 63×, and z-stack images were acquired. Image settings were kept consistent for all samples. Maximum intensity projections (MIPs) were created using the ImageJ distribution Fiji (53). To quantify virus entry, MIPs were analyzed using CellProfiler v4.1.3 (54). Total VLPs were identified using the Alexa Fluor 488 signal, and a mask was generated in the Alexa Fluor 568 channel to include Alexa Fluor 488–positive objects only. Within this mask, Alexa Fluor 568 signal was used to identify the extracellular virus. Counts were summarized using pivot tables, and virus uptake was quantified by comparing the Alexa Fluor 568 signal to the total Alexa Fluor 488 signal in each sample. Uptake was expressed as the percentage of internalized particles in entry samples and binding controls. Statistical analysis of the fold increase was performed using Welch’s *t* test. Due to the very low signal in samples with only VLP, GCA, CA, and GCDCA, these conditions were omitted for uptake quantification. The number of cells was determined by manual counting from MIPs. More than 70 cells and at least 3 fields of view per condition were imaged and analyzed. Size quantification of signal clusters was performed by identifying VLPs as objects by the Alexa Fluor 488 signal in all MIPs and subsequently quantifying all Alexa Fluor 488 signal by number and signal size using Cell Profiler v4.1.3. Quantification of differentially sized clusters was done by adjusting the lower size limit of pixels (where pixel size was 180 nm/px) detected to 0px, 5px, and 15px to separate three cluster sizes, while keeping the upper limit to a maximum of 35 pixels in diameter, which captured the largest signal clusters. Statistical analysis of the increase compared to the VLP control was done using the mixed effects model with the Geisser-Greenhouse correction.

### Electron microscopy

Bile acids TLCA, GCDCA, GCA, CA, or human bile were mixed with 15 µg HuNoV GII.4 VLPs in 20 mM HEPES buffer pH 8.2 to a final concentration of 250 µM or 4 mM, followed by 30-min incubation at 37°C. For negative staining electron microscopy (EM), copper grids were coated with thin layers of formvar and carbon (sputtered with Leica EM ACE 200), and glow-discharged (PELCO easiGlow). 3.5 µL VLP samples were applied to each grid, blotted to filter paper, washed with two drops of 50 µL H_2_0, and stained with a 50 µL drop of 1.5% Uranyl Acetate on parafilm. Stained VLPs were imaged with a Talos L120 transmission electron microscope (TEM, Thermo Fisher Scientific, former FEI) with 47,000× magnification. Micrographs were acquired with a Ceta 4k × 4k CMOS detector using Velox software (Thermo Fisher Scientific, former FEI).

### Statistical analysis

All experiments were performed three times in duplicates or triplicates. The results are shown as mean ± SD. Two-way analysis of variance (ANOVA), Student’s *t* test, Welch’s *t* test, or mixed model analysis was performed using GraphPad Prism version 9.3 for Windows (GraphPad Software, San Diego, California, USA). A *P*-value of <0.05 was considered statistically significant.

## RESULTS

### HuNoV VLPs can bind to human duodenal cells in the presence of human bile or hydrophobic bile acids

**Fig 1:**
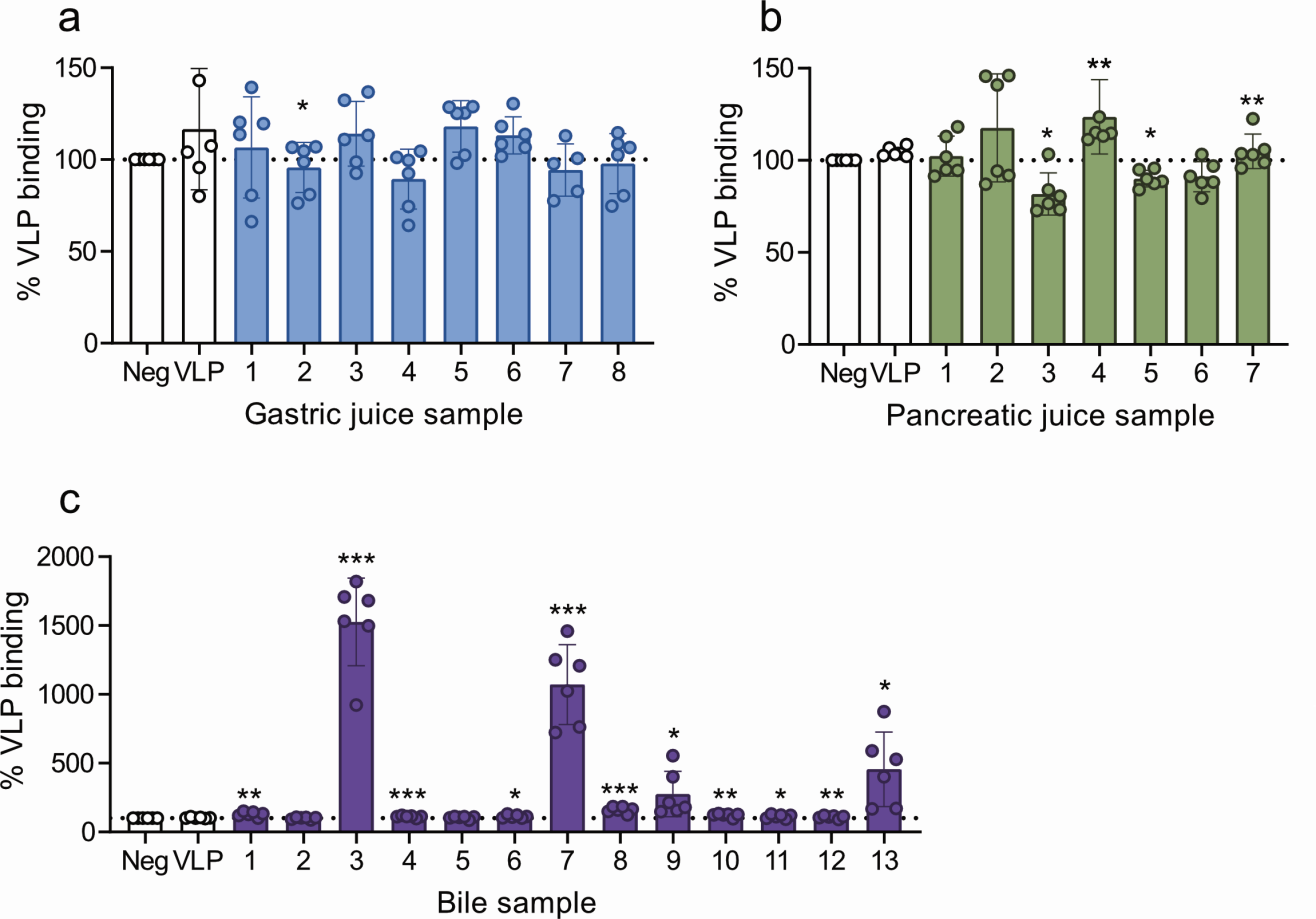
Bile, but not gastric juice or pancreatic juice, enhances GII.4 VLP binding to HuTu-80 cells. VLP binding to HuTu-80 cells in the presence of gastric juice (**a**), pancreatic juice (**b**), or bile (**c**) samples from patients. Binding was measured as median fluorescence and presented as a percentage of control (VLP binding in the absence of human gastrointestinal fluids). Data were generated from three independent experiments, represented as means ± SD. Statistical significance was determined with Student’s *t* test; *, *P* < 0.05; **, *P* < 0.002; ***, *P* < 0.001.

**Fig 2:**
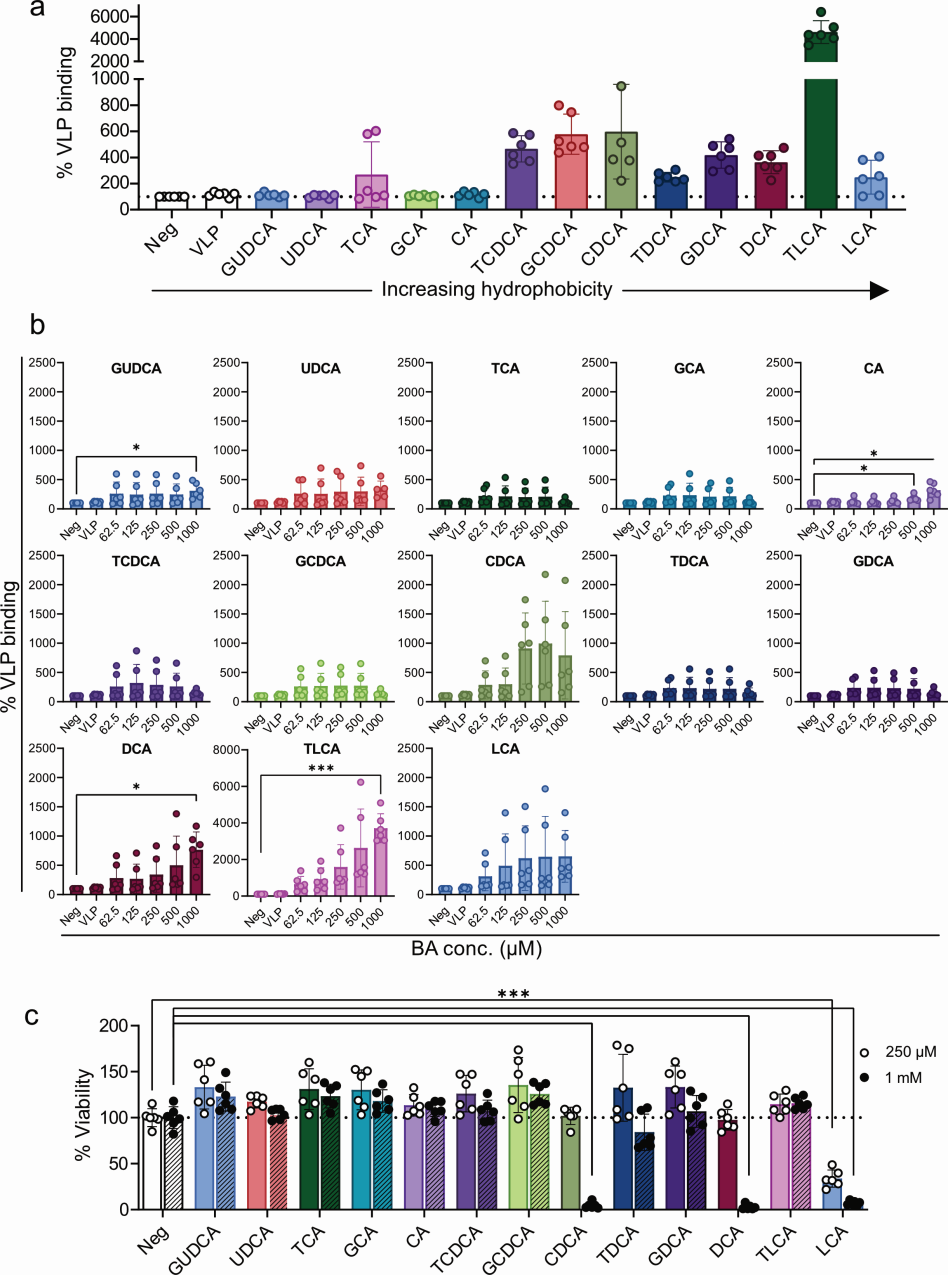
Hydrophobic bile acids enhance GII.4 VLP binding to HuTu-80 cells. (**a**) VLP binding to HuTu-80 cells in the presence of bile acids at a concentration of 250 µM, with bile acids arranged by hydrophobicity. (**b**) VLP binding to HuTu-80 cells with a titration of bile acids ranging from 62.5 to 1000 µM. (**c**) Viability of HuTu-80 cells in the presence of bile acids at 250 µM and 1 mM. Binding was measured as median fluorescence and presented as a percentage of control (VLP binding in the absence of bile acids). Cell viability was assessed by ATP production using Celltiter Glo (Promega). Data are from three independent experiments, represented as means ± SD. For binding, statistical significance was determined with Student’s *t* test; *, *P* < 0.05; **, *P* < 0.002; ***, *P* < 0.001. For cell viability, statistical significance was determined with two-way ANOVA; ***, *P* < 0.001.

It has been demonstrated that bile can enhance replication of HuNoV GII.3 and GII.4 variants (5) and that bile acids increase cell surface ceramide expression, resulting in enhanced viral uptake (42). Since bile acids can also interact directly with the HuNoV capsid, we investigated whether human gastrointestinal fluids, including gastric juice, pancreatic juice, and bile, affect the direct attachment of GII.4 VLPs to a human duodenal adenocarcinoma cell line (HuTu-80). VLP binding was unaffected by gastric and pancreatic juice samples (Fig. 1a and b) but was largely enhanced by some, but not all, bile samples (Fig. 1c). As human bile composition varies between individuals in both amounts and types of bile acids, we investigated whether individual bile acids directly affect VLP binding to cells by pre-incubating VLPs with bile acids and quantifying the binding to HuTu-80 cells. At 250 µM, several bile acids increased binding (Fig. 2a), among them glycochenodeoxycholic acid (GCDCA), which has previously been associated with increased HuNoV replication in enteroids (42). We also observed a 30-fold increase in attachment mediated by the highly hydrophobic taurine-conjugated bile acid TLCA (Fig. 2a). These results are consistent with previous findings, showing that HuNoV prefers hydrophobic bile acids for cell uptake and replication (42). Bile acid concentrations are highest proximal to the bile ducts and decrease toward the distal small intestine. In addition, concentrations rise transiently following food intake. The concentration of 250 µM used in the initial analysis is within the physiological range (55–57). Dose-response analysis of bile acids (62.5 µM–1000 µM, two-fold increments) showed enhanced VLP binding up to 500 µM for several bile acids, with a reduced effect at 1,000 µM (TCA, GCA, TCDCA, GCDCA, and GDCA), while others showed enhancing properties also at 1,000 µM (CA, DCA, TLCA, and LCA) (Fig. 2b). Next, we assessed the viability of HuTu-80 cells after bile acid treatment to determine whether the observed effects on virus binding could be attributed to cytotoxicity. At 250 µM, only LCA exhibited toxicity, and at 1,000 µM, CDCA and DCA showed toxic effects (Fig. 2c).

### Bile acids exert a direct effect on HuNoV VLP binding to cells

**Fig 3:**
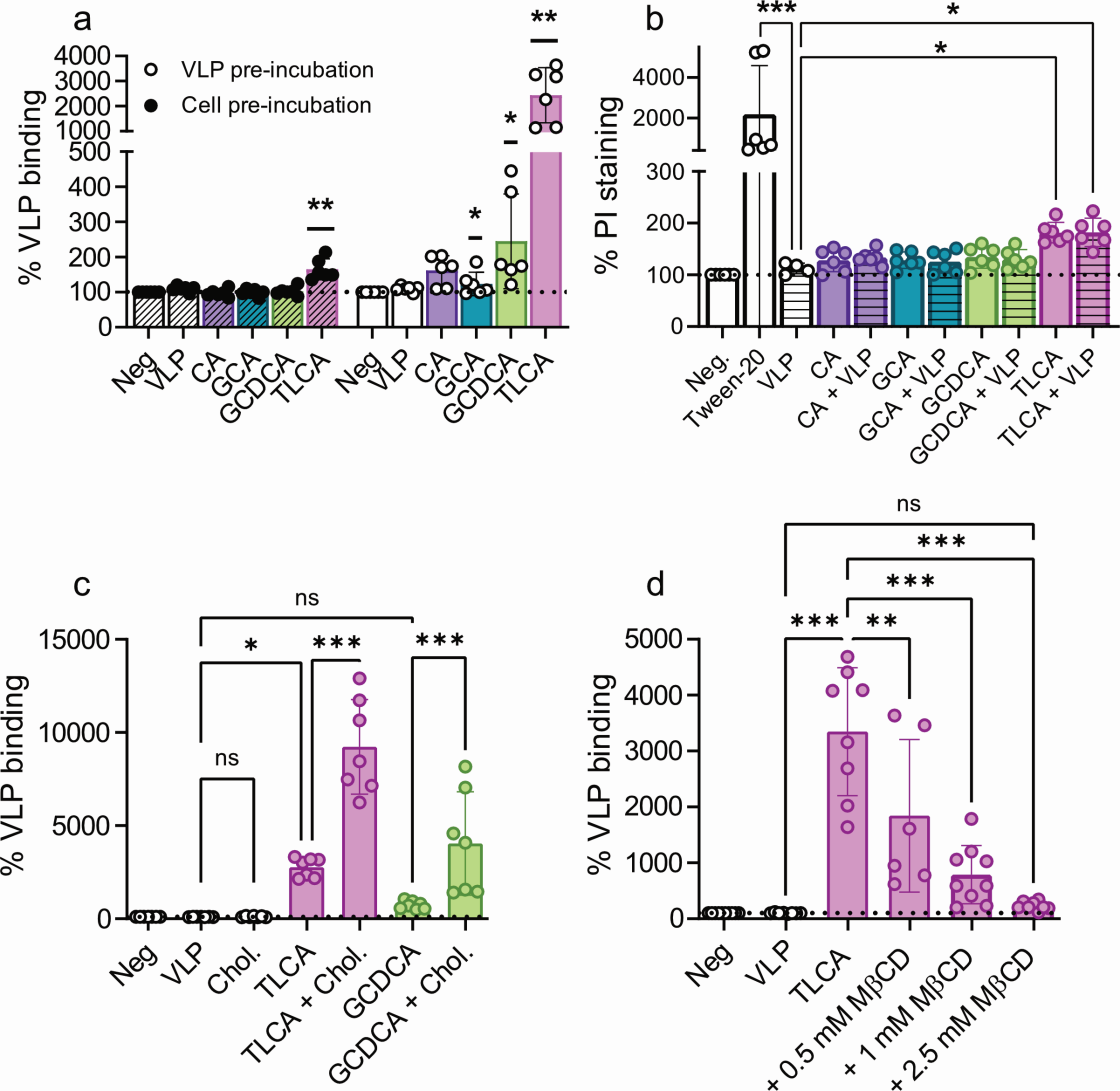
Bile acid-mediated GII.4 VLP binding to HuTu-80 cells is enhanced by cholesterol. (**a**) VLP binding to HuTu-80 cells in the presence of bile acids, where bile acids were either pre-incubated with cells before the addition of VLPs (Cell pre-treatment), or pre-incubated with VLPs before the addition of cells (VLP pre-incubation). (**b**) Cell permeabilization in the presence of bile acids or bile acids + VLPs. Tween-20 was used as a positive control. (**c**) VLP binding to HuTu-80 cells after preincubation with TLCA, GCDCA, cholesterol, or a combination of cholesterol and TLCA/GCDCA, respectively. (**d**) VLP binding to HuTu-80 cells in the presence of TLCA, after cell pre-treatment with cholesterol sequestrant MβCD. Binding was measured as median fluorescence and presented as a percentage of control (VLP binding in the absence of bile acids). Data are from three independent experiments, represented as means ± SD. Statistical significance was determined with two-way ANOVA; *, *P* < 0.05; ***, *P* < 0.001.

Subsequently, we investigated whether bile acid-mediated VLP binding resulted from cellular effects or direct interactions with the VLPs. To test this, we pre-treated either cells or VLPs with bile acids prior to the binding assay. While TLCA increased binding in both conditions, the most prominent increase was seen when bile acids were pre-incubated with VLPs (Fig. 3a). These results suggest that the enhanced VLP binding observed in Fig. 2a is not due to bile acid-induced changes in the target cell but rather requires a direct interaction between bile acids and VLPs. As bile acids are amphipathic molecules, a property shared with detergents, we assessed their effect on cell permeability. We incubated cells with bile acids alone or with bile acids and VLPs and assessed cell permeability with propidium iodide staining. A minor increase in cell permeability was observed with TLCA and TLCA+VLPs, whereas no effect was seen with the other bile acids (Fig. 3b). We then pre-incubated VLPs with cholesterol as it is known to mediate entry and replication for many viruses, including HuNoV GII.3 and GII.4 (31, 42, 58, 59). Alone, cholesterol had no effect on binding, but when combined with TLCA or GCDCA, cholesterol significantly enhanced the bile acid-mediated VLP binding (Fig. 3c), suggesting it has an amplifying effect. Furthermore, removing cell membrane cholesterol by pre-treatment with the cholesterol sequestrant methyl-β-cyclodextrin (MβCD) reduced TLCA-mediated VLP binding in a dose-dependent manner, where at 2.5 mM MβCD, the binding was completely abolished (Fig. 3d). Taken together, these results show that bile acids directly enhance VLP binding to cells, and that the presence of other hydrophobic molecules such as cholesterol further enhances this effect.

### Affinity measurements confirm direct interaction between GII.4 VLPs and TLCA

To confirm direct interactions between bile acids and VLPs, surface plasmon resonance (SPR) was used to measure binding affinity. VLPs were immobilized on the sensor chip as ligand, and bile acids flowed over the surface as analytes. The resulting sensograms showed rapid association and dissociation kinetics for all bile acids with detectable binding, indicating transient interactions. Among the tested bile acids, TLCA exhibited the strongest binding to VLPs with an estimated affinity of 64 µM, followed by GCDCA at 123 µM (Table 1). In contrast, neither GCA nor CA showed any association with VLPs, and no affinity could be determined for these compounds (Table 1).

**TABLE 1:** SPR shows direct interactions between bile acids and GII.4 VLPs^*a*^

Ligand	Analyte	K_D_ (µM) ± SD	*n*
GII.4 VLP	TDCA	308 ± 29.7	2
GII.4 VLP	GDCA	286.5 ± 64.3	2
GII.4 VLP	TCDCA	236 ± 58	2
GII.4 VLP	DCA	176.5 ± 4.9	2
GII.4 VLP	GCDCA	123 ± 24	2
GII.4 VLP	TLCA	64 ± 9.9	2
GII.4 VLP	GCA	NB	2
GII.4 VLP	CA	NB	2

^*a*^ SPR results, expressed as mean K_D_, shows affinity between GII.4 VLPs and bile acids. The experiment was performed twice. CA and GCA were non-binding, displayed as NB.

### HBGA-independent GII.1 VLPs can also use TLCA and bile for increased binding

**Fig 4:**
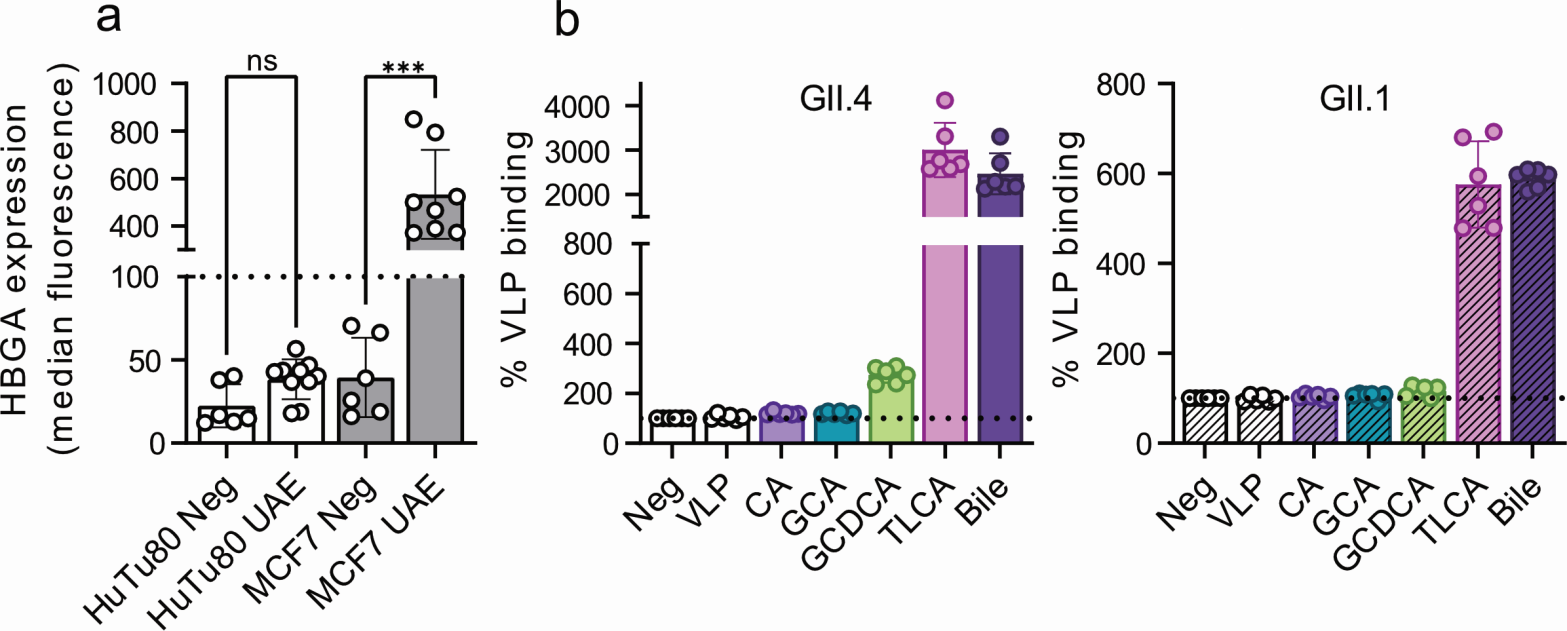
HBGA-dependent GII.1 VLPs show increased binding to HuTu-80 cells, despite low HBGA cell expression. (**a**) HBGA expression on HuTu-80 cells. MCF-7 cells were used as a positive control for HBGA expression. (**b**) Cell binding with GII.4 and GII.1 in the presence of bile and bile acids. Data are from three independent experiments, represented as means ± SD. Statistical significance was determined with two-way ANOVA; ***, *P* < 0.001.

As GII.4 is an HBGA-dependent HuNoV genotype, we tested whether our HuTu-80 cells express HBGAs. Compared to the HBGA-positive MCF7 cell line, HuTu-80 cells showed no detectable HBGA expression (Fig. 4a). Notably, as GII.4 VLPs were still able to bind HuTu-80 cells in the presence of TLCA, this suggests that the TLCA-mediated binding we observe does not require HBGAs. To explore whether this mechanism also applies to HBGA-independent HuNoV strains, we performed binding experiments using GII.1 (HV strain) VLPs. GII.1 VLPs were incubated with GCA, CA, GCDCA, TLCA, or bile, and compared to GII.4 binding under the same conditions. Like GII.4, GII.1 VLPs showed enhanced binding to HuTu-80 cells in the presence of TLCA and bile, but not with GCDCA (Fig. 4b).

### Bile acids are required for HuNoV VLP entry in HuTu-80 cells

**Fig 5:**
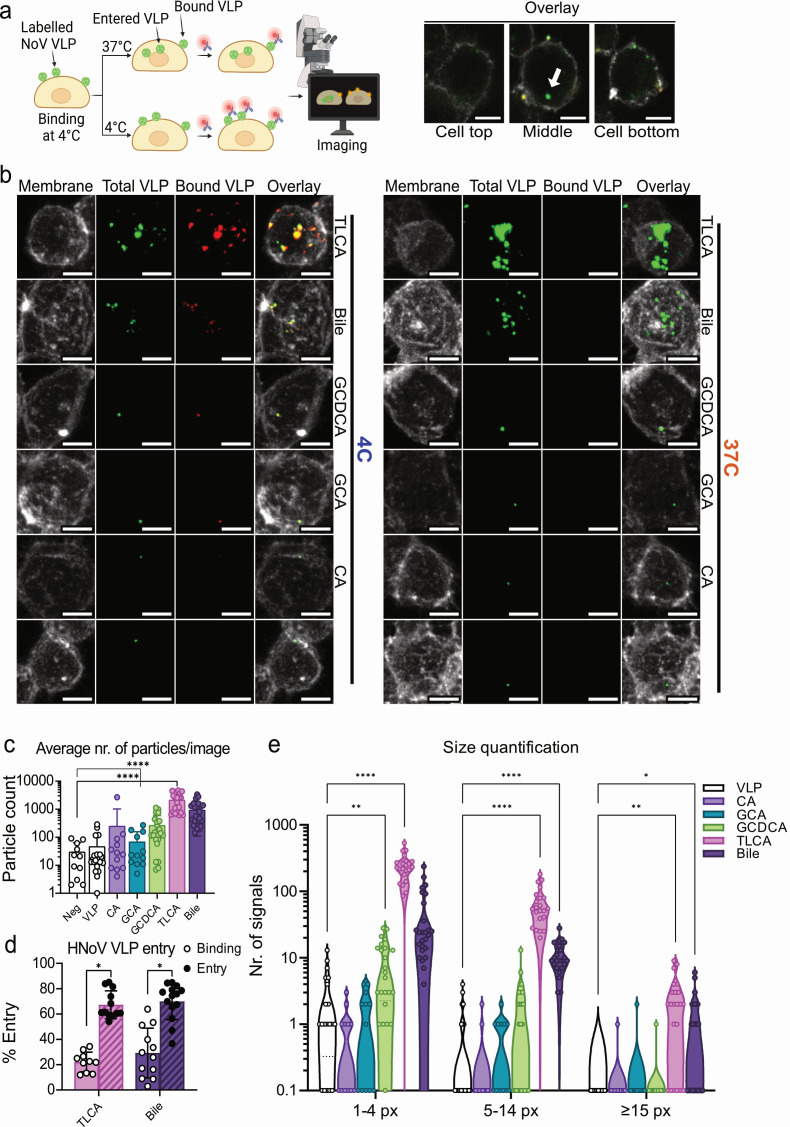
TLCA aggregates and enhances the entry of GII.4 VLP into HuTu-80 cells. (**a**) (Left) Experimental design: VLPs, labeled with Alexa Fluor 488, were pre-incubated with bile or bile acids before addition to cells, after which they were allowed to bind (4°C) and subsequently enter cells (37°C). Remaining cell-bound particles were stained with an anti-Alexa Fluor 488 antibody and a secondary Alexa Fluor 568 antibody. The cell membrane was stained with wheat germ agglutinin. (Right) Representative z-stack slices of one cell at increasing z-stack depth, visualizing VLP signal appearing inside the cell (white arrow), never outside of the cell membrane. (**b**) Cells were visualized using a Leica SP8 laser scanning confocal microscope and depicted as representative maximum intensity projections of VLP + TLCA, VLP + human bile sample 3, VLP + GCDCA, VLP + GCA, VLP + CA, and only VLPs at 4°C (binding control) and 37°C (entry). Scale bar for all images is 5.4 microns. (**c**) Virus binding was increased in the presence of bile or bile acid, assessed by object count of Alexa Fluor 488 signal, which was measured for all images and displayed as the average number of objects per image. As the average number of objects per image for samples with only VLPs, GCA + VLP, CA + VLP, and GCDCA + VLP was very low, these were excluded from uptake quantification. (**d**) Virus entry was quantified by measuring all virus particles (AF488) and extracellular virus particles (AF568) and presented as a relative comparison between the extracellular and all virus object counts in each sample. (**e**) Signal clusters were quantified by measuring the number of signal clusters and gradually excluding smaller clusters by increasing the minimum amount of pixel size for each image, where pixel size was 180 nm/px. Statistical significance was determined with (c) Kruskal-Wallis test; *, *P* < 0.05; **, *P* < 0.002; ***, *P* < 0.001; (d) multiple Mann-Whitney test; and (e) mixed model method for quantification of size clusters, where * indicates a significant increase compared to only the VLP sample. The experiment was repeated two times, with at least three representative images captured for each experiment.

As bile acids increased the binding of GII.4 VLPs to HuTu-80 cells, we next investigated whether they also promote VLP entry. GCDCA can indirectly increase the entry of GII.3 in HIEs through cellular effects, such as changes in endosomal acidification and apical membrane ceramide levels (42). However, it remains unclear whether other bile acids, or human bile itself, can directly promote VLP entry. We investigated this by examining both binding and entry in the presence of bile acids using laser scanning confocal microscopy. Alexa Fluor (AF)−488 (green) labeled GII.4 VLPs, pre-incubated with bile acids, were added to HuTu-80 cells on ice to allow surface binding, followed by a 1-hour incubation at 37°C to permit internalization. Live, non-permeabilized cells were stained with an anti-AF488 primary antibody followed by an AF568 (red)-conjugated secondary antibody to allow distinction between surface-bound VLPs (dual-labeled, appearing yellow), and internalized VLPs (green only) (Fig. 5a [left]). Both single-stained internalized particles (green) and double-stained bound particles at the cell membrane (yellow) could be visualized in the images (as exemplified in Fig. 5a [right]). We found almost no binding or entry of VLPs in the absence of bile acids, or with bile acids that did not increase binding (GCA and CA) as seen by low AF488 signal (Fig. 5b). Surprisingly, no increase in binding or entry was observed with GCDCA in confocal images, despite enhanced binding detected with flow cytometry (Fig. 2a). In contrast, incubation with human bile or TLCA led to clear increases in both surface-bound (increased external yellow signal) and internalized (increased green signal) VLPs (Fig. 5b). We then ascertained VLP entry by careful analysis of single cells at variable z-stack planes and used these images to perform a relative comparison of total VLP signal in the absence or presence of bile or bile acids. In the absence, or with the bile acids GCA, CA, and GCDCA, very low VLP signal was detected, which was not significantly increased compared to the negative control (Fig. 5c). We next quantified VLP uptake for the remaining conditions and found that bile, and more so TLCA, significantly (*P* < 0.001 for both bile and TLCA) increased VLP uptake (Fig. 5d), in line with signal levels seen in the images (Fig. 5b). Notably, we also observed a bile acid-dependent increase in total fluorescence intensity and the appearance of larger signal accumulations. This was most prominent in the bile and TLCA samples. Quantification of signal clusters, separated by size, confirmed a marked increase in large clusters with TLCA and bile, in contrast to non-binding bile acids or VLPs alone (Fig. 5e). Taken together, these results suggest that TLCA and bile mediate direct binding to and subsequent entry of HuNoV VLPs into HuTu-80 cells, and that they also induce clustering of VLPs.

### Bile acids and bile aggregate norovirus-like particles

**Fig 6:**
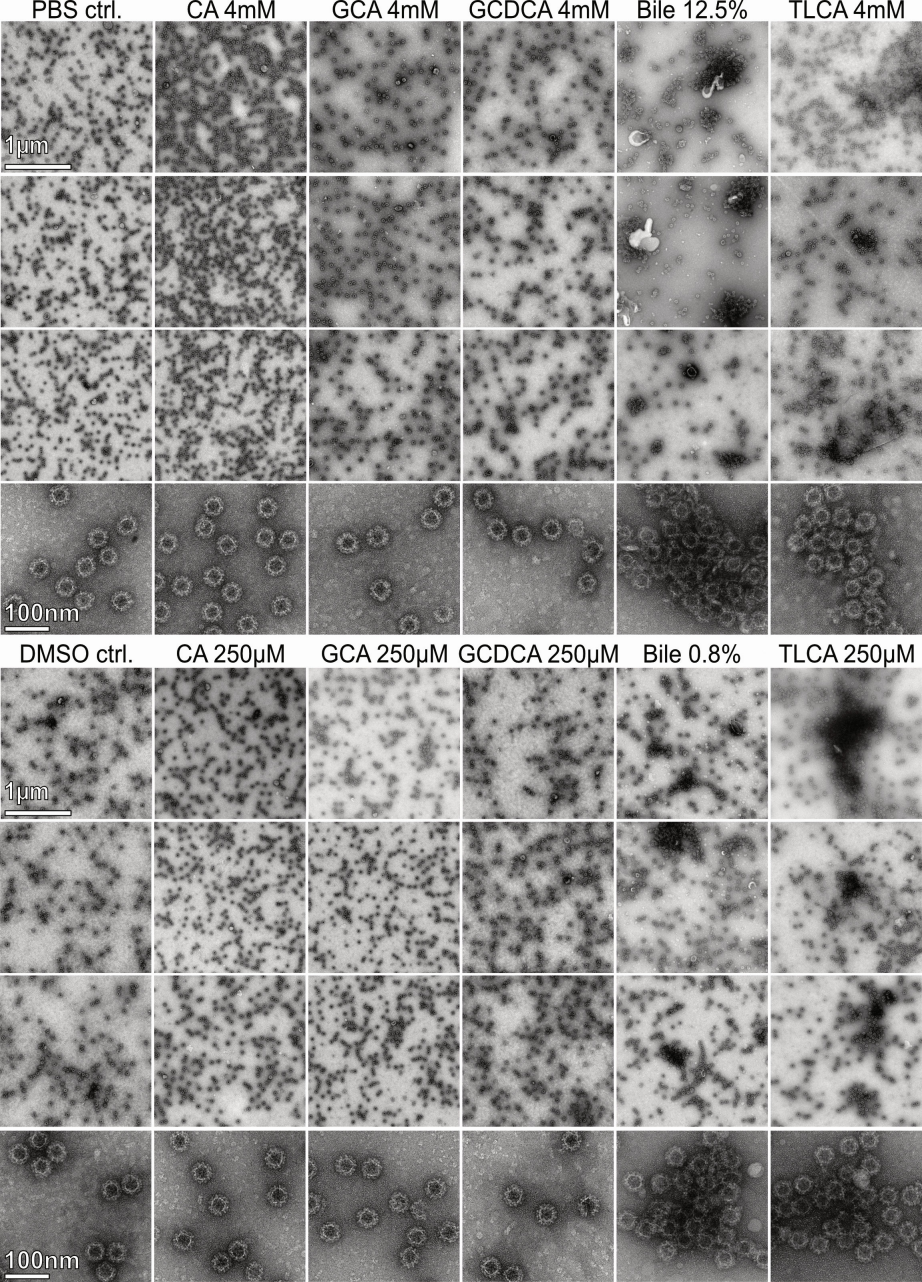
GII.4 VLPs cluster in the presence of TLCA and bile, but not GCDCA, GCA, or CA. HuNoV GII.4 VLPs imaged with electron microscopy, either in the presence of bile acids TLCA, GCDCA, GCA, or CA at concentrations of 4 mM (upper panels) or 250 µM (lower panels) or in the presence of human bile at a concentration of 12.5% (upper panels) or 0.8% (lower panels). VLPs incubated in only PBS or DMSO served as control samples. Shown are three representative images, captured with 13,500× magnification, as well as one representative image captured with 73,000× magnification. Scale bars are shown in PBS control and DMSO control images and are 1 µM and 100 nm, respectively.

The signal accumulation observed by confocal microscopy suggests aggregation of VLPs, possibly resulting from interactions with the hydrophobic bile acids. To investigate this hypothesis, we performed EM to visualize VLPs incubated with increasing concentrations of bile or bile acids. VLPs without bile or bile acids showed an even distribution of particles on the grid with only small clusters with few VLPs (Fig. 6). Similarly, the bile acids GCA and CA, which displayed no effect on either binding or uptake (Fig. 2a and 5e), showed no clustering. The addition of the bile acid GCDCA did not visibly alter the distribution, and no large clusters were observed at either the pre-incubation concentration (4 mM) or the binding concentration (250 µM) (Fig. 6). However, in the presence of TLCA or human bile, we observed a marked difference in VLP distribution. At 4 mM (pre-incubation condition), TLCA caused formation of large clusters consisting of several dozen VLPs (Fig. 6), and even at the lower binding concentration of 250 µM, TLCA induced considerable accumulations of 10 or more VLPs (Fig. 6). We used bile from patient number 3, as this sample yielded the largest increase in VLP binding to HuTu-80 cells (Fig. 1c). In the presence of 12.5% bile (pre-incubation concentration) and 0.8% bile (binding concentration), multiple VLPs clustered to a size similar to that observed with TLCA (Fig. 6). VLP clusters after bile treatment resembled those seen with TLCA more than those formed with GCDCA. As TLCA is more hydrophobic than GCDCA, this particular bile sample could contain higher levels of hydrophobic bile acids or components. These results suggest that TLCA, a highly hydrophobic bile acid, promotes cellular attachment and VLP uptake by inducing aggregation of HuNoV VLPs.

## DISCUSSION

We demonstrate that bile acids mediate direct HuNoV GII.4 VLP binding to and entry into a human duodenal cell line, HuTu-80. This complements the knowledge that bile acids exert indirect effects to overcome the entry restrictions of HuNoV replication in human enteroids. Additionally, we observe accumulations of VLPs upon incubation with human bile and the hydrophobic bile acid TLCA. Quantification of VLP uptake in cells further suggests that clustering does not inhibit viral entry but rather increases it. Recently, it has been shown that GII.4 VLPs accumulate on the cell surface of HIEs in a HBGA-dependent manner, leading to receptor clustering, increased viral entry, and cell membrane wounding (50). The same study also shows that disruption of membrane cholesterol inhibits binding and entry, which is in line with our findings that treatment with MβCD dose-dependently reduces the effect of TLCA. It has previously been shown that HuNoV GII.4 causes cell permeability in human intestinal enteroids, but only if there is an initial interaction with HBGAs (31). Yet, in our setup using HuTu-80 cells, we did not observe increased cell permeability when bile acids or VLPs were present.

*In vivo*, bile acids form micelles in the human intestine which aid in both excretion and absorption (39). We speculate that TLCA and other hydrophobic bile acids, possibly in conjunction with cholesterol, provide a platform for aggregation of viral particles. We propose that aggregates may enter host cells by similar mechanisms as single particles, or by novel, hitherto unknown mechanisms. It has been shown that enteric viruses such as rotavirus and HuNoV are secreted in extracellular vesicles, and that these vesicle-cloaked viral clusters enhances both infection efficiency and disease severity (48, 49, 60–63). Whether vesicle-mediated entry bypasses receptor requirements appears to vary by virus. For example, the binding of MNoV 1, hepatitis A virus, and poliovirus, released within extracellular vesicles, is blocked by antibodies against their respective receptors CD300lf, TIM-1, and CD155 (48, 49, 62). In other cases, such as JC polyomavirus, vesicle-mediated infectivity is receptor-independent, which can explain why the virus becomes more permissive *in vivo* (63). Additionally, the virus can be fully or partially released from vesicles in proximity to the cell surface, or vesicles can express markers that target them to receptor-expressing tissues, as speculated by Santiana et al. about MNoV and Chen et al. regarding poliovirus (48, 49).

We show that hydrophobic bile acids are the most efficient enhancers of viral binding and entry and that other hydrophobic molecules, such as cholesterol, add to this effect through an indirect mechanism that is not yet characterized. These results are consistent with previous studies showing that cholesterol increases replication of GII.3 and GII.4. The average human bile pool only contains trace amounts of highly hydrophobic bile acids such as TLCA due to their cytotoxicity at higher concentrations (39, 64). However, human bile does not only contain bile acids but also amphiphilic phospholipids and cholesterol (among other components) that together form mixed micelles in the duodenum (65). The total bile acid concentration at which micelles are formed is around 4 mM, but the concentration can reach up to 15 mM after ingestion of food (66, 67). The strong, additive effect of cholesterol, together with TLCA-mediated binding, indicates that even a small amount of TLCA within a micelle could be enough to facilitate binding and uptake of HuNoV. We further show that the removal of cholesterol from the cell membrane with the sequestrant MβCD completely abolishes the effect of TLCA, suggesting that cell membrane-associated cholesterol can enhance bile acid-mediated interactions. It can, however, not mediate VLP binding on its own, which parallels previous work showing that detergents (with properties similar to bile acids) do not mediate increased HuNoV replication (42). Thus, the increased binding and entry cannot be explained by hydrophobic interactions alone and show that bile acids seem to have unique features that makes them pro-viral for HuNoV. In a recent study, GII.4 replication in FUT2-expressing HIEs was inhibited by MβCD treatment, despite the fact that no bile acids were present (31), suggesting that cholesterol is important for multiple receptor interaction mechanisms. It is therefore tempting to speculate that receptor clustering by cholesterol-mediated lipid raft formation is also true for HuNoV virus-host interaction.

Using SPR, we established direct interaction between VLPs and TLCA with a K_D_ of 64 µM. However, fast association and dissociation would indicate that it is transitional in nature. Comparative studies have determined the K_D_ between HuNoV and HBGAs to be 2–30 mM (68), indicating that VLP interactions with bile acids are stronger, or at least comparable, to those with HBGAs. Still, SPR is optimal for measuring affinities between ligands and analytes of a similar size. Thus, our measurement of bile acids binding to VLPs, where the analyte is smaller than the ligand, can result in reduced signal-to-noise ratio and lower response unit (69). In our binding experiments, we used the HBGA-negative cell line HuTu-80. Although GII.4 is an HBGA-dependent genotype, secretor-negative individuals can seemingly still be infected by HBGA-dependent HuNoVs (28–30), suggesting the existence of alternative mechanisms for attachment. One such mechanism may involve bile acids; in fact, a bile acid-binding pocket has been identified in the exposed P-domain of GII.4 (44). Additionally, bile acid treatment has been shown to confer HBGA-binding capacity on the otherwise HBGA-independent GII.1 genotype (43). We also observed enhanced binding of GII.1 VLPs to HuTu-80 cells in the presence of bile acids, supporting a role for bile acids as factors during HuNoV attachment.

As a cancer-derived cell line, HuTu-80 may not fully recapitulate the physiological environment of the small intestines. However, as a duodenal epithelial model, they have been widely used to investigate intestinal transport, epithelial receptor function, and enteric virus infection, including bile acid–dependent replication of human sapovirus, another calicivirus (70–72). These findings indicate that key epithelial membrane properties and endocytic pathways relevant to virus attachment and entry are preserved in this model.

In conclusion, we show that bile acids mediate direct attachment and entry of HuNoV GII.4 VLPs. We suggest that this mechanism is associated with the ability of bile to accumulate VLPs, which could be of relevance as other intestinal viruses also benefit from cell entry in clusters for infection. Our results also indicate that other tissue fluid compounds, such as cholesterol, enhance the effect of bile acids on HuNoV attachment and cell entry. Studying the properties of micelles isolated from HuNoV-susceptible individuals might elucidate whether single bile acids like TLCA, or the overall bile acid composition, is important for increased binding and uptake. Taken together, our findings provide valuable knowledge regarding HuNoV biology and add to the understanding of attachment and entry of one of the most common causes of viral gastroenteritis.

## Data Availability

All data pertaining to this study are presented in the article.

## References

[1] Bartsch SM, Lopman BA, Ozawa S, Hall AJ, Lee BY. 2016. Global economic burden of norovirus gastroenteritis. PLoS One 11:e0151219. doi:10.1371/journal.pone.0151219 27115736 PMC4846012

[2] Chhabra P, de Graaf M, Parra GI, Chan MC-W, Green K, Martella V, Wang Q, White PA, Katayama K, Vennema H, Koopmans MPG, Vinjé J. 2019. Updated classification of norovirus genogroups and genotypes. J Gen Virol 100:1393–1406. doi:10.1099/jgv.0.001318 31483239 PMC7011714

[3] Jones MK, Watanabe M, Zhu S, Graves CL, Keyes LR, Grau KR, Gonzalez-Hernandez MB, Iovine NM, Wobus CE, Vinjé J, Tibbetts SA, Wallet SM, Karst SM. 2014. Enteric bacteria promote human and mouse norovirus infection of B cells. Science 346:755–759. doi:10.1126/science.1257147 25378626 PMC4401463

[4] Kroneman A, Verhoef L, Harris J, Vennema H, Duizer E, van Duynhoven Y, Gray J, Iturriza M, Böttiger B, Falkenhorst G, et al.. 2008. Analysis of integrated virological and epidemiological reports of norovirus outbreaks collected within the foodborne viruses in Europe network from 1 July 2001 to 30 June 2006. J Clin Microbiol 46:2959–2965. doi:10.1128/JCM.00499-08 18650354 PMC2546741

[5] Ettayebi K, Crawford SE, Murakami K, Broughman JR, Karandikar U, Tenge VR, Neill FH, Blutt SE, Zeng X-L, Qu L, Kou B, Opekun AR, Burrin D, Graham DY, Ramani S, Atmar RL, Estes MK. 2016. Replication of human noroviruses in stem cell–derived human enteroids. Science 353:1387–1393. doi:10.1126/science.aaf5211 27562956 PMC5305121

[6] Hoa Tran TN, Trainor E, Nakagomi T, Cunliffe NA, Nakagomi O. 2013. Molecular epidemiology of noroviruses associated with acute sporadic gastroenteritis in children: global distribution of genogroups, genotypes and GII.4 variants. J Clin Virol 56:185–193. doi:10.1016/j.jcv.2012.11.011 23218993

[7] Prasad BV, Matson DO, Smith AW. 1994. Three-dimensional structure of calicivirus. J Mol Biol 240:256–264. doi:10.1006/jmbi.1994.1439 8028008

[8] Prasad BV, Hardy ME, Dokland T, Bella J, Rossmann MG, Estes MK. 1999. X-ray crystallographic structure of the Norwalk virus capsid. Science 286:287–290. doi:10.1126/science.286.5438.287 10514371

[9] Taube S, Rubin JR, Katpally U, Smith TJ, Kendall A, Stuckey JA, Wobus CE. 2010. High-resolution X-ray structure and functional analysis of the murine norovirus 1 capsid protein protruding domain. J Virol 84:5695–5705. doi:10.1128/JVI.00316-10 20335262 PMC2876589

[10] Jiang X, Wang M, Graham DY, Estes MK. 1992. Expression, self-assembly, and antigenicity of the Norwalk virus capsid protein. J Virol 66:6527–6532. doi:10.1128/JVI.66.11.6527-6532.1992 1328679 PMC240146

[11] Duizer E, Schwab KJ, Neill FH, Atmar RL, Koopmans MPG, Estes MK. 2004. Laboratory efforts to cultivate noroviruses. J Gen Virol 85:79–87. doi:10.1099/vir.0.19478-0 14718622

[12] Lay MK, Atmar RL, Guix S, Bharadwaj U, He H, Neill FH, Sastry KJ, Yao Q, Estes MK. 2010. Norwalk virus does not replicate in human macrophages or dendritic cells derived from the peripheral blood of susceptible humans. Virology (Auckl) 406:1–11. doi:10.1016/j.virol.2010.07.001 PMC2933743 20667573

[13] Straub TM, Höner zu Bentrup K, Orosz-Coghlan P, Dohnalkova A, Mayer BK, Bartholomew RA, Valdez CO, Bruckner-Lea CJ, Gerba CP, Abbaszadegan M, Nickerson CA. 2007. In vitro cell culture infectivity assay for human noroviruses. Emerg Infect Dis 13:396–403. doi:10.3201/eid1303.060549 17552092 PMC2725917

[14] Guix S, Asanaka M, Katayama K, Crawford SE, Neill FH, Atmar RL, Estes MK. 2007. Norwalk virus RNA is infectious in mammalian cells. J Virol 81:12238–12248. doi:10.1128/JVI.01489-07 17855551 PMC2168986

[15] Oka T, Stoltzfus GT, Zhu C, Jung K, Wang Q, Saif LJ. 2018. Attempts to grow human noroviruses, a sapovirus, and a bovine norovirus in vitro. PLoS One 13:e0178157. doi:10.1371/journal.pone.0178157 29438433 PMC5810978

[16] Marionneau S, Ruvoën N, Le Moullac-Vaidye B, Clement M, Cailleau-Thomas A, Ruiz-Palacois G, Huang P, Jiang X, Le Pendu J. 2002. Norwalk virus binds to histo-blood group antigens present on gastroduodenal epithelial cells of secretor individuals. Gastroenterology 122:1967–1977. doi:10.1053/gast.2002.33661 12055602 PMC7172544

[17] Harrington PR, Vinjé J, Moe CL, Baric RS. 2004. Norovirus capture with histo-blood group antigens reveals novel virus-ligand interactions. J Virol 78:3035–3045. doi:10.1128/jvi.78.6.3035-3045.2004 14990722 PMC353760

[18] Atmar RL, Ramani S, Estes MK. 2018. Human noroviruses: recent advances in a 50-year history. Curr Opin Infect Dis 31:422–432. doi:10.1097/QCO.0000000000000476 30102614

[19] Kahan SM, Liu G, Reinhard MK, Hsu CC, Livingston RS, Karst SM. 2011. Comparative murine norovirus studies reveal a lack of correlation between intestinal virus titers and enteric pathology. Virology (Auckl) 421:202–210. doi:10.1016/j.virol.2011.09.030 PMC3210872 22018636

[20] Roth AN, Helm EW, Mirabelli C, Kirsche E, Smith JC, Eurell LB, Ghosh S, Altan-Bonnet N, Wobus CE, Karst SM. 2020. Norovirus infection causes acute self-resolving diarrhea in wild-type neonatal mice. Nat Commun 11:2968. doi:10.1038/s41467-020-16798-1 32528015 PMC7289885

[21] Peiper AM, Helm EW, Nguyen Q, Phillips M, Williams CG, Shah D, Tatum S, Iyer N, Grodzki M, Eurell LB, Nasir A, Baldridge MT, Karst SM. 2023. Infection of neonatal mice with the murine norovirus strain WU23 is a robust model to study norovirus pathogenesis. Lab Anim (NY) 52:119–129. doi:10.1038/s41684-023-01166-5 37142696 PMC10234811

[22] Donaldson EF, Lindesmith LC, Lobue AD, Baric RS. 2010. Viral shape-shifting: norovirus evasion of the human immune system. Nat Rev Microbiol 8:231–241. doi:10.1038/nrmicro2296 20125087 PMC7097584

[23] Lindesmith L, Moe C, Marionneau S, Ruvoen N, Jiang X, Lindblad L, Stewart P, LePendu J, Baric R. 2003. Human susceptibility and resistance to norwalk virus infection. Nat Med 9:548–553. doi:10.1038/nm860 12692541

[24] Huang P, Farkas T, Marionneau S, Zhong W, Ruvoën-Clouet N, Morrow AL, Altaye M, Pickering LK, Newburg DS, LePendu J, Jiang X. 2003. Noroviruses bind to human ABO, lewis, and secretor histo-blood group antigens: identification of 4 distinct strain-specific patterns. J Infect Dis 188:19–31. doi:10.1086/375742 12825167

[25] Haga K, Ettayebi K, Tenge VR, Karandikar UC, Lewis MA, Lin SC, Neill FH, Ayyar BV, Zeng XL, Larson G, Ramani S, Atmar RL, Estes MK. 2020. Genetic manipulation of human intestinal enteroids demonstrates the necessity of a functional fucosyltransferase 2 gene for secretor-dependent human norovirus infection. mBio 11:e00251-20. doi:10.1128/mBio.00251-20 32184242 PMC7078471

[26] Tan M, Huang P, Meller J, Zhong W, Farkas T, Jiang X. 2003. Mutations within the P2 domain of norovirus capsid affect binding to human histo-blood group antigens: evidence for a binding pocket. J Virol 77:12562–12571. doi:10.1128/jvi.77.23.12562-12571.2003 14610179 PMC262557

[27] Tan M, Hegde RS, Jiang X. 2004. The P domain of norovirus capsid protein forms dimer and binds to histo-blood group antigen receptors. J Virol 78:6233–6242. doi:10.1128/JVI.78.12.6233-6242.2004 15163716 PMC416535

[28] Nordgren J, Kindberg E, Lindgren PE, Matussek A, Svensson L. 2010. Norovirus gastroenteritis outbreak with a secretor-independent susceptibility pattern, Sweden. Emerg Infect Dis 16:81–87. doi:10.3201/eid1601.090633 20031047 PMC2874438

[29] de Rougemont A, Ruvoen-Clouet N, Simon B, Estienney M, Elie-Caille C, Aho S, Pothier P, Le Pendu J, Boireau W, Belliot G. 2011. Qualitative and quantitative analysis of the binding of GII.4 norovirus variants onto human blood group antigens. J Virol 85:4057–4070. doi:10.1128/JVI.02077-10 21345963 PMC3126233

[30] Carlsson B, Kindberg E, Buesa J, Rydell GE, Lidón MF, Montava R, Abu Mallouh R, Grahn A, Rodríguez-Díaz J, Bellido J, Arnedo A, Larson G, Svensson L. 2009. The G428A nonsense mutation in FUT2 provides strong but not absolute protection against symptomatic GII.4 norovirus infection. PLoS One 4:e5593. doi:10.1371/journal.pone.0005593 19440360 PMC2680586

[31] Ayyar BV, Ettayebi K, Salmen W, Karandikar UC, Neill FH, Tenge VR, Crawford SE, Bieberich E, Prasad BVV, Atmar RL, Estes MK. 2023. CLIC and membrane wound repair pathways enable pandemic norovirus entry and infection. Nat Commun 14:1148. doi:10.1038/s41467-023-36398-z 36854760 PMC9974061

[32] Johansson C, Jonsson M, Marttila M, Persson D, Fan XL, Skog J, Frängsmyr L, Wadell G, Arnberg N. 2007. Adenoviruses use lactoferrin as a bridge for CAR-independent binding to and infection of epithelial cells. J Virol 81:954–963. doi:10.1128/JVI.01995-06 17079302 PMC1797453

[33] Persson BD, Lenman A, Frängsmyr L, Schmid M, Ahlm C, Plückthun A, Jenssen H, Arnberg N. 2020. Lactoferrin-hexon interactions mediate CAR-independent adenovirus infection of human respiratory cells. J Virol 94:e00542-20. doi:10.1128/JVI.00542-20 32376620 PMC7343212

[34] Kalyuzhniy O, Di Paolo NC, Silvestry M, Hofherr SE, Barry MA, Stewart PL, Shayakhmetov DM. 2008. Adenovirus serotype 5 hexon is critical for virus infection of hepatocytes in vivo. Proc Natl Acad Sci USA 105:5483–5488. doi:10.1073/pnas.0711757105 18391209 PMC2291105

[35] Klenk HD, Rott R, Orlich M, Blödorn J. 1975. Activation of influenza A viruses by trypsin treatment. Virology (Auckl) 68:426–439. doi:10.1016/0042-6822(75)90284-6 173078

[36] Le TQ, Kawachi M, Yamada H, Shiota M, Okumura Y, Kido H. 2006. Identification of trypsin I as a candidate for influenza A virus and Sendai virus envelope glycoprotein processing protease in rat brain. Biol Chem 387:467–475. doi:10.1515/BC.2006.062 16606346

[37] Ghosh S, Kumar M, Santiana M, Mishra A, Zhang M, Labayo H, Chibly AM, Nakamura H, Tanaka T, Henderson W, Lewis E, Voss O, Su Y, Belkaid Y, Chiorini JA, Hoffman MP, Altan-Bonnet N. 2022. Enteric viruses replicate in salivary glands and infect through saliva. Nature 607:345–350. doi:10.1038/s41586-022-04895-8 35768512 PMC9243862

[38] Boyer JL. 2013. Bile formation and secretion. Compr Physiol 3:1035–1078. doi:10.1002/cphy.c120027 23897680 PMC4091928

[39] Monte MJ, Marin JJG, Antelo A, Vazquez-Tato J. 2009. Bile acids: chemistry, physiology, and pathophysiology. World J Gastroenterol 15:804–816. doi:10.3748/wjg.15.804 19230041 PMC2653380

[40] Russell DW. 2003. The enzymes, regulation, and genetics of bile acid synthesis. Annu Rev Biochem 72:137–174. doi:10.1146/annurev.biochem.72.121801.161712 12543708

[41] Chang KO, Sosnovtsev SV, Belliot G, Kim Y, Saif LJ, Green KY. 2004. Bile acids are essential for porcine enteric calicivirus replication in association with down-regulation of signal transducer and activator of transcription 1. Proc Natl Acad Sci USA 101:8733–8738. doi:10.1073/pnas.0401126101 15161971 PMC423264

[42] Murakami K, Tenge VR, Karandikar UC, Lin SC, Ramani S, Ettayebi K, Crawford SE, Zeng XL, Neill FH, Ayyar BV, Katayama K, Graham DY, Bieberich E, Atmar RL, Estes MK. 2020. Bile acids and ceramide overcome the entry restriction for GII.3 human norovirus replication in human intestinal enteroids. Proc Natl Acad Sci USA 117:1700–1710. doi:10.1073/pnas.1910138117 31896578 PMC6983410

[43] Kilic T, Koromyslova A, Hansman GS. 2019. Structural basis for human norovirus capsid binding to bile acids. J Virol 93:e01581-18. doi:10.1128/JVI.01581-18 30355683 PMC6321941

[44] Creutznacher R, Schulze E, Wallmann G, Peters T, Stein M, Mallagaray A. 2020. Chemical-shift perturbations reflect bile acid binding to norovirus coat protein: recognition comes in different flavors. Chembiochem 21:1007–1021. doi:10.1002/cbic.201900572 31644826 PMC7186840

[45] Sherman MB, Williams AN, Smith HQ, Nelson C, Wilen CB, Fremont DH, Virgin HW, Smith TJ. 2019. Bile salts alter the mouse norovirus capsid conformation: possible implications for cell attachment and immune evasion. J Virol 93:e00970-19. doi:10.1128/JVI.00970-19 31341042 PMC6744230

[46] Nelson CA, Wilen CB, Dai YN, Orchard RC, Kim AS, Stegeman RA, Hsieh LL, Smith TJ, Virgin HW, Fremont DH. 2018. Structural basis for murine norovirus engagement of bile acids and the CD300lf receptor. Proc Natl Acad Sci USA 115:E9201–E9210. doi:10.1073/pnas.1805797115 30194229 PMC6166816

[47] Williams AN, Sherman MB, Smith HQ, Taube S, Pettitt BM, Wobus CE, Smith TJ. 2021. A norovirus uses bile salts to escape antibody recognition while enhancing receptor binding. J Virol 95:e0017621. doi:10.1128/JVI.00176-21 33827952 PMC8315966

[48] Santiana M, Ghosh S, Ho BA, Rajasekaran V, Du W-L, Mutsafi Y, De Jésus-Diaz DA, Sosnovtsev SV, Levenson EA, Parra GI, Takvorian PM, Cali A, Bleck C, Vlasova AN, Saif LJ, Patton JT, Lopalco P, Corcelli A, Green KY, Altan-Bonnet N. 2018. Vesicle-cloaked virus clusters are optimal units for inter-organismal viral transmission. Cell Host Microbe 24:208–220. doi:10.1016/j.chom.2018.07.006 30092198 PMC6226266

[49] Chen YH, Du W, Hagemeijer MC, Takvorian PM, Pau C, Cali A, Brantner CA, Stempinski ES, Connelly PS, Ma HC, Jiang P, Wimmer E, Altan-Bonnet G, Altan-Bonnet N. 2015. Phosphatidylserine vesicles enable efficient en bloc transmission of enteroviruses. Cell 160:619–630. doi:10.1016/j.cell.2015.01.032 25679758 PMC6704014

[50] Ayyar BV, Apostol CV, Dave JJ, Kaundal S, Kendra JA, Neill FH, Ettayebi K, Maher S, Anish R, Parra GI, Larson G, Atmar RL, Crawford SE, Prasad BVV, Estes MK. 2025. Functional diversity in GII.4 norovirus entry: HBGA binding and capsid clustering dynamics. Proc Natl Acad Sci USA 122:e2517493122. doi:10.1073/pnas.2517493122 41032521 PMC12519127

[51] Lindesmith LC, Debbink K, Swanstrom J, Vinjé J, Costantini V, Baric RS, Donaldson EF. 2012. Monoclonal antibody-based antigenic mapping of norovirus GII.4-2002. J Virol 86:873–883. doi:10.1128/JVI.06200-11 22090098 PMC3255811

[52] Chen CL, Jensen RL, Schnepp BC, Connell MJ, Shell R, Sferra TJ, Bartlett JS, Clark KR, Johnson PR. 2005. Molecular characterization of adeno-associated viruses infecting children. J Virol 79:14781–14792. doi:10.1128/JVI.79.23.14781-14792.2005 16282478 PMC1287571

[53] Schindelin J, Arganda-Carreras I, Frise E, Kaynig V, Longair M, Pietzsch T, Preibisch S, Rueden C, Saalfeld S, Schmid B, Tinevez JY, White DJ, Hartenstein V, Eliceiri K, Tomancak P, Cardona A. 2012. Fiji: an open-source platform for biological-image analysis. Nat Methods 9:676–682. doi:10.1038/nmeth.2019 22743772 PMC3855844

[54] Stirling DR, Swain-Bowden MJ, Lucas AM, Carpenter AE, Cimini BA, Goodman A. 2021. CellProfiler 4: improvements in speed, utility and usability. BMC Bioinformatics 22:433. doi:10.1186/s12859-021-04344-9 34507520 PMC8431850

[55] Bajor A, Gillberg PG, Abrahamsson H. 2010. Bile acids: short and long term effects in the intestine. Scand J Gastroenterol 45:645–664. doi:10.3109/00365521003702734 20334475

[56] Lenci I, Milana M, Signorello A, Grassi G, Baiocchi L. 2023. Secondary bile acids and the biliary epithelia: the good and the bad. World J Gastroenterol 29:357–366. doi:10.3748/wjg.v29.i2.357 36687129 PMC9846939

[57] Hofmann AF. 1999. The continuing importance of bile acids in liver and intestinal disease. Arch Intern Med 159:2647–2658. doi:10.1001/archinte.159.22.2647 10597755

[58] Gerondopoulos A, Jackson T, Monaghan P, Doyle N, Roberts LO. 2010. Murine norovirus-1 cell entry is mediated through a non-clathrin-, non-caveolae-, dynamin- and cholesterol-dependent pathway. J Gen Virol 91:1428–1438. doi:10.1099/vir.0.016717-0 20147520

[59] Martín JJ, Holguera J, Sánchez-Felipe L, Villar E, Muñoz-Barroso I. 2012. Cholesterol dependence of newcastle disease virus entry. Biochim Biophys Acta 1818:753–761. doi:10.1016/j.bbamem.2011.12.004 22192779 PMC7094422

[60] Cuevas JM, Durán-Moreno M, Sanjuán R. 2017. Multi-virion infectious units arise from free viral particles in an enveloped virus. Nat Microbiol 2:17078. doi:10.1038/nmicrobiol.2017.78 28530650 PMC5447809

[61] Baez-Navarro C, Quevedo IR, López S, Arias CF, Iša P. 2022. The association of human astrovirus with extracellular vesicles facilitates cell infection and protects the virus from neutralizing antibodies. J Virol 96:e0084822. doi:10.1128/jvi.00848-22 35762754 PMC9327681

[62] Feng Z, Hensley L, McKnight KL, Hu F, Madden V, Ping L, Jeong SH, Walker C, Lanford RE, Lemon SM. 2013. A pathogenic picornavirus acquires an envelope by hijacking cellular membranes. Nature 496:367–371. doi:10.1038/nature12029 23542590 PMC3631468

[63] Morris-Love J, Gee GV, O’Hara BA, Assetta B, Atkinson AL, Dugan AS, Haley SA, Atwood WJ. 2019. JC polyomavirus uses extracellular vesicles to infect target cells. mBio 10:e00379-19. doi:10.1128/mBio.00379-19 30967463 PMC6456752

[64] Begley M, Gahan CGM, Hill C. 2005. The interaction between bacteria and bile. FEMS Microbiol Rev 29:625–651. doi:10.1016/j.femsre.2004.09.003 16102595

[65] Hofmann AF. 1999. Bile acids: the good, the bad, and the ugly. News Physiol Sci 14:24–29. doi:10.1152/physiologyonline.1999.14.1.24 11390813

[66] Humbert L, Rainteau D, Tuvignon N, Wolf C, Seksik P, Laugier R, Carrière F. 2018. Postprandial bile acid levels in intestine and plasma reveal altered biliary circulation in chronic pancreatitis patients. J Lipid Res 59:2202–2213. doi:10.1194/jlr.M084830 30206181 PMC6210915

[67] Amara S, Bourlieu C, Humbert L, Rainteau D, Carrière F. 2019. Variations in gastrointestinal lipases, pH and bile acid levels with food intake, age and diseases: Possible impact on oral lipid-based drug delivery systems. Adv Drug Deliv Rev 142:3–15. doi:10.1016/j.addr.2019.03.005 30926476

[68] Peters T, Creutznacher R, Maass T, Mallagaray A, Ogrissek P, Taube S, Thiede L, Uetrecht C. 2022. Norovirus-glycan interactions - how strong are they really? Biochem Soc Trans 50:347–359. doi:10.1042/BST20210526 34940787 PMC9022987

[69] Sparks RP, Jenkins JL, Fratti R. 2019. Use of surface plasmon resonance (SPR) to determine binding affinities and kinetic parameters between components important in fusion machinery. Methods Mol Biol 1860:199–210. doi:10.1007/978-1-4939-8760-3_12 30317506 PMC8489108

[70] Oka T, Li TC, Yonemitsu K, Ami Y, Suzaki Y, Kataoka M, Doan YH, Okemoto-Nakamura Y, Kobayashi T, Saito H, Mita T, Tokuoka E, Shibata S, Yoshida T, Takagi H. 2024. Propagating and banking genetically diverse human sapovirus strains using a human duodenal cell line: investigating antigenic differences between strains. J Virol 98:e0063924. doi:10.1128/jvi.00639-24 39132992 PMC11406923

[71] Oka T, Okemoto-Nakamura Y, Takagi H. 2025. CD36 is required for human sapovirus propagation. J Virol 99:e0132525. doi:10.1128/jvi.01325-25 41190813 PMC12646008

[72] Takagi H, Oka T, Shimoike T, Saito H, Kobayashi T, Takahashi T, Tatsumi C, Kataoka M, Wang Q, Saif LJ, Noda M. 2020. Human sapovirus propagation in human cell lines supplemented with bile acids. Proc Natl Acad Sci USA 117:32078–32085. doi:10.1073/pnas.2007310117 33257564 PMC7749338

